# Masked polycythemia vera due to iron deficiency anemia

**DOI:** 10.1002/ccr3.7157

**Published:** 2023-03-30

**Authors:** Koma Hotta, Kiyoshi Shikino, Ryotaro Niwa

**Affiliations:** ^1^ Department of General Internal Medicine Takatsuki Hospital Osaka Japan; ^2^ Department of General Medicine China University Hospital Chiba Japan; ^3^ Department of Hematology Japanese Red Cross Takatsuki Hospital Osaka Japan

**Keywords:** helicobacter pylori, iron deficiency anemia, palmar erythema, polycythemia vera

## Abstract

A 74‐year‐old Japanese woman was referred to our hospital for leukocytosis that occurred for the past one year. Oral iron supplementation was started as iron deficiency anemia (IDA), but three months later, physical examination revealed flushing of the skin on her hands. Finally polycythemia vera (PV) with IDA was diagnosed. There have been reports of PV combined with IDA, which can mask diagnosis and delay treatment because of the lack of symptoms and the anemic presentation. Several possibilities for the pathogenesis of IDA associated with PV have been proposed, including the presence of Helicobacter pylori.

## Abstract 2

In this case, palmar erythema appeared during the treatment of iron deficiency anemia (IDA), which eventually led to the diagnosis of masked polycythemia vera (PV) due to IDA.

In August 2021, a 74‐year‐old Japanese woman was referred to our hospital due to leukocytosis that had occurred in the past years. The patient denied pruritus, visual disturbances, or palmar erythema. Her complete blood count parameters were as follows: white blood cells, 14,600/μL; red blood cells, 5.66 × 10^6^/μL; hemoglobin, 10.5 g/dL; mean corpuscular volume, 63.3 fL; platelets, 739 × 10^3^/μL; ferritin, 3.1 ng/mL. Upper gastrointestinal endoscopy revealed atrophic gastritis with *Helicobacter pylori*, and colonoscopy revealed no abnormal findings. We suspected iron deficiency anemia (IDA) and initiated oral iron supplementation. In November 2021, hemoglobin levels improved to 16.4 g/dL, but leukocytosis and thrombocytosis remained unchanged. Physical examination revealed flushing of the skin on her hands without pain or dysesthesia (Figure 1). Additional laboratory tests showed erythropoietin levels of 0.7 mIU/mL (reference value 4.2–23.7), vitamin B12 levels of 1490 pg/mL, and a mutation in the novel Janus kinase 2 (JAK2) gene. Abdominal ultrasonography revealed splenomegaly (112 mm × 67 mm). She was then referred to a hematologist, and a bone marrow biopsy revealed hyperplasia with megakaryocytes. A diagnosis of polycythemia vera (PV) with IDA was made. Hydroxyurea, phlebotomy, and aspirin were continued for PV treatment.

**FIGURE 1:**
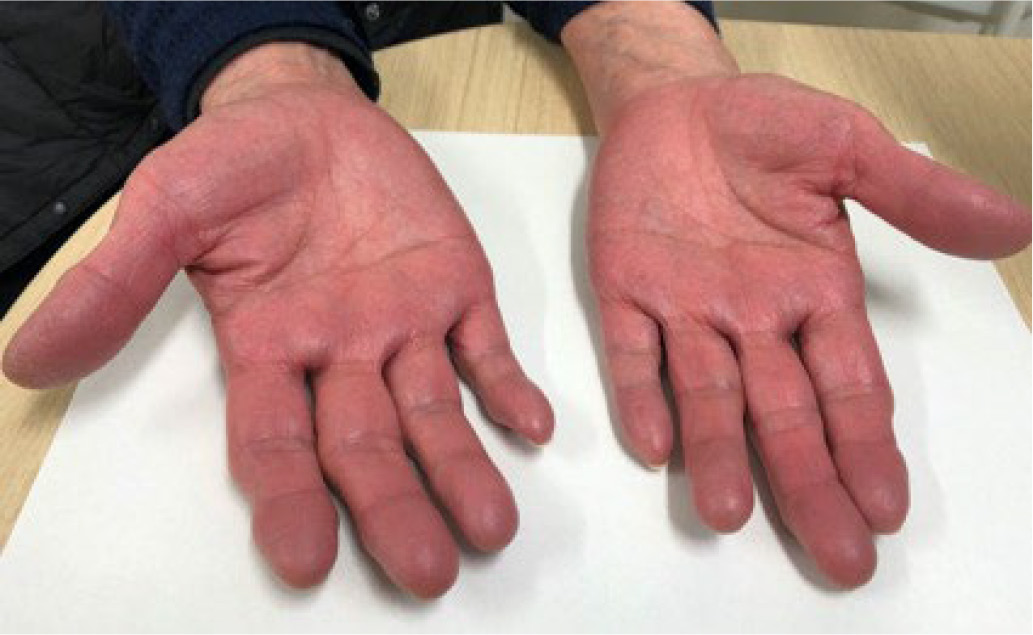
Palmar erythema after oral iron supplementation.

PV is one of the myeloproliferative neoplasms characterized by an increased red cell mass on normal hemoglobin oxygen saturation. Affected individuals may have elevated white blood cell and platelet counts. This condition is characterized by fatigue, pruritus, and an increased risk of thrombosis.

Chronic leukocytosis has been observed in this patient for several years. PV can be associated with leukocytosis in some cases.^1^ Since the patient had a history of smoking 10 cigarettes per day, PV and smoking were considered possible causes of leukocytosis. Compensatory leukocytosis due to IDA was also considered but was ruled out as the leukocytosis persisted even after iron supplementation improved the anemia.

There have been reports of PV combined with IDA, which can mask diagnosis and delay treatment because of the anemic presentation. Several possibilities for the pathogenesis of IDA associated with PV have been proposed as follows^2^: decreased expression of erythroferrone (ERFE), activation of JAK/STAT, decreased iron absorption, or complications of gastrointestinal bleeding. Gastroduodenal lesions and the presence of *Helicobacter pylori* are more commonly found in patients with PV than in the general population^3^ and may be related to gastrointestinal bleeding and decreased iron absorption. In the present case, the cause of IDA was thought to be a decrease in iron absorption by *Helicobacter pylori*.

In the case of PV combined with IDA, the diagnosis may be delayed owing to the lack of symptoms, and the presence of anemia in blood tests. In this case, palmar erythema appeared during the treatment of iron deficiency anemia, eventually leading to the PV diagnosis masked by IDA. Careful physical examination is one of the keys to the diagnosis.

## AUTHOR CONTRIBUTIONS

KH cared for the patient and wrote the first draft. KS and RN read and approved the final version of the report. All authors had access to the data and a role in writing the manuscript.

## FUNDING INFORMATION

None.

## CONFLICT OF INTEREST STATEMENT

None.

## CONSENT FOR PUBLICATION

Written informed consent was obtained from the patient to publish this report in accordance with the journal's patient consent policy.

## Data Availability

Data sharing is not applicable to this article, as no datasets were generated or analyzed during the current study.
